# Genetic Analysis of NBS-LRR Gene Family in Chickpea and Their Expression Profiles in Response to Ascochyta Blight Infection

**DOI:** 10.3389/fpls.2017.00838

**Published:** 2017-05-19

**Authors:** Mandeep S. Sagi, Amit A. Deokar, Bunyamin Tar’an

**Affiliations:** Department of Plant Sciences, College of Agriculture and Bioresources, University of Saskatchewan, SaskatoonSK, Canada

**Keywords:** NBS-LRR genes, expression profiling, ascochyta blight, chickpea

## Abstract

Ascochyta blight is one of the major diseases of chickpea worldwide. The genetic resistance to ascochyta blight in chickpea is complex and governed by multiple QTLs. However, the molecular mechanism of quantitative disease resistance to ascochyta blight and the genes underlying these QTLs are still unknown. Most often disease resistance is determined by resistance (R) genes. The most predominant R-genes contain nucleotide binding site and leucine rich repeat (NBS-LRR) domains. A total of 121 NBS-LRR genes were identified in the chickpea genome. Ninety-eight of these genes contained all essential conserved domains while 23 genes were truncated. The NBS-LRR genes were grouped into eight distinct classes based on their domain architecture. Phylogenetic analysis grouped these genes into two major clusters based on their structural variation, the first cluster with toll or interleukin-1 like receptor (TIR) domain and the second cluster either with or without a coiled-coil domain. The NBS-LRR genes are distributed unevenly across the eight chickpea chromosomes and nearly 50% of the genes are present in clusters. Thirty of the NBS-LRR genes were co-localized with nine of the previously reported ascochyta blight QTLs and were tested as potential candidate genes for ascochyta blight resistance. Expression pattern of these genes was studied in two resistant (CDC Corinne and CDC Luna) and one susceptible (ICCV 96029) genotypes at different time points after ascochyta blight infection using real-time quantitative PCR. Twenty-seven NBS-LRR genes showed differential expression in response to ascochyta blight infection in at least one genotype at one time point. Among these 27 genes, the majority of the NBS-LRR genes showed differential expression after inoculation in both resistant and susceptible genotypes which indicates the involvement of these genes in response to ascochyta blight infection. Five NBS-LRR genes showed genotype specific expression. Our study provides a new insight of NBS-LRR gene family in chickpea and the potential involvement of NBS-LRR genes in response to ascochyta blight infection.

## Introduction

Plant pathogens are diverse in nature which include bacteria, virus, fungi, and nematodes. Each of them deploys various approaches to draw nutrition from plants such as biotrophy, necrotrophy, or hemi-biotrophy. The necrotrophic fungi are well known to have broad host ranges and can cause severe economic losses in agriculture (Pusztahelyi et al., 2015). Plants and pathogens have co-evolved together and each has adapted different survival strategies. Plant pathogens have evolved the ability to invade plants, suppress plant defense response and colonize plant tissue for their growth and reproduction. To cope with the wide array of pathogens, plants have developed a sophisticated immune system (Hammond-Kosack and Jones, 1997; Qi and Innes, 2013). Plant immune system differs from vertebrate animals, as plants lack adaptive immune system. Alternatively, plants solely rely on its bi-layered cell-autonomous immune system to perceive and respond to the invading pathogens (Jones and Dangl, 2006). Most pathogen infections are prevented by non-host resistance conferred by the first layer of plant basal defense response which is triggered by recognition of pathogen-associated molecular patterns (PAMPs) by the plant pattern recognition receptors (PRRs) localized on the plasma membrane. Some specifically adapted pathogens can overcome the first barrier by delivering effector proteins into plant cells to suppress the host basal defense. Such host-specific pathogens are countered by the second layer of defense termed as effector-triggered immunity (ETI) mediated by the intracellular receptors encoded by the plant disease resistance gene (R-genes) that recognize the presence of pathogen effector protein and activate downstream immune responses to inhibit pathogen infection.

Several classes of R-genes have been identified and classified based on their putative protein domain organization and their localization in the plant cell (van Ooijen et al., 2007). The most abundant class of R-genes is characterized by the encoding proteins that consist of central Nucleotide Binding Site (NBS) and carboxyl/C-terminal Leucine Rich Repeat (LRR) domain, hence called NBS-LRR genes (McHale et al., 2006). Plant NBS-LRR proteins are mainly intracellular receptors that can perceive the presence of pathogen effector directly by binding to the pathogen effector proteins or indirectly by recognition of any modification in the pathogen effector target proteins in host and activate multiple defense signal transductions which often result in hypersensitive response and other biochemical changes that limit pathogen growth (Meyers et al., 2003; DeYoung and Innes, 2006). The central NBS domain (also known as NB-ARC domain) is composed of strictly ordered conserved motifs which are required for ATP and GTP binding and hydrolysis. Plant NBS domain shows structural homology between Apaf-1 and CED4 domains that are involved in animal cell apoptosis, which suggests similarity in their modes of action (Dangl and Jones, 2001; DeYoung and Innes, 2006). The NBS domain is followed by several tandem LRRs which are known to provide recognition specificity toward pathogen effector molecules (Innes, 2004).

The plant NBS-LRR genes can be classified into two sub-classes based on the presence or absence of amino/N-terminal domain. The first sub-class comprises proteins that carry *Drosophila* Toll and INTERLEUKIN1 like receptor (TIR) domain at the N-terminal position and are termed as TIR-NBS-LRR (TNL). The other sub-class comprises proteins which often carry Coiled-coil (CC) domain and are known as CC-NBS-LRR (CNL; Meyers et al., 1999). Other domains such as Zinc Finger or RPW8 domain are also found in the N-terminal position instead of CC domain which is often classified under CNL class (Sukarta et al., 2016). Recent studies demonstrated the function of TIR and CC domain in pathogen recognition and downstream signaling (Maekawa et al., 2011; Williams et al., 2014). The distribution of TNL and CNL gene classes is species specific as dicots contain both classes while monocots lack the TNL class (Shao et al., 2016).

Chickpea (*Cicer arietinum* L.) is a self-pollinated diploid crop with genome size of 738 Mb and the world’s second most cultivated food legume crop after dry bean (FAOSTAT, 2015). Chickpea production is vital for food security and for improving the nutritional quality of diets in many developing countries particularly in South Asia, where it serves as a staple for human protein. Global chickpea production has increased by 56% in the last decade (2004–2013; Gaur et al., 2016). Both biotic and abiotic stresses are the major challenges of chickpea production limiting the crop to express its maximum yield potential. Ascochyta blight caused by the necrotrophic fungus *Ascochyta rabiei* (Pass) Labrousse is one of the major diseases of chickpea worldwide which lowers both grain yield and grain quality.

*Ascochyta rabiei* infects all above ground parts of chickpea plants which can result in total crop loss if favorable environmental conditions prevail for its infection and further growth. Ascochyta blight is a major concern as most of the cultivated chickpea accessions are susceptible to the disease, which limits the efforts to breed for ascochyta blight resistant cultivars. Genetic resistance to ascochyta blight is a complex trait and is highly influenced by environmental conditions. To date, several quantitative trait loci (QTLs) for ascochyta blight resistance have been identified in diverse genetic backgrounds on linkage groups (LGs) 2, 3, 4, 6 and 8 (Santra et al., 2000; Flandez-Galvez et al., 2003; Cho et al., 2004; Iruela et al., 2006; Tar’an et al., 2007; Anbessa et al., 2009; Sabbavarapu et al., 2013; Stephens et al., 2014). Most of these QTLs were tagged with SSR markers. The genes underlying the resistance are still unknown. So far two candidate genes CaETR-1 (EIN-4 like) and *ethylene insensitive 3-like gene* (*Ein3*) from ethylene pathway were identified in ascochyta blight resistance QTL_AR1_ on LG4 and QTL_AR3_ on LG2, respectively (Madrid et al., 2012, 2014). These studies suggest the possible involvement of ethylene pathway in ascochyta blight resistance in chickpea. In a recent study, expression profiling of 15 defense-related genes in response to *Ascochyta rabiei* infection identified six differentially expressed genes among ten chickpea genotypes (Leo et al., 2016). Identification of candidate genes involved in resistance to ascochyta blight associated QTLs will help in understanding the resistance mechanism and further assists in the development of resistant cultivars using marker assisted selection.

Homologs of NBS-LRR genes have been identified in *Arabidopsis* (Meyers et al., 2003), rice (Monosi et al., 2004), *Medicago* (Ameline-Torregrosa et al., 2008), cassava (Lozano et al., 2015), soybean (Kang et al., 2012), *Brassica* (Mun et al., 2009), potato (Lozano et al., 2012) and in many other plant species. Most studies identified variable numbers of NBS-LRR genes ranging from 50 in papaya (Porter et al., 2009) to 1,015 in apple (Arya et al., 2014). The NBS-LRR genes are unevenly distributed in plant genomes and are often found in clusters. The availability of the draft genome sequence of chickpeas (Jain et al., 2013; Varshney et al., 2013) provided an opportunity to explore genome-wide distribution of several gene families such as Aux/IAA gene family (Singh and Jain, 2015), F-box genes (Gupta et al., 2015), ERF genes (Deokar et al., 2015), CaNAC genes (Ha et al., 2014), UDP-glycosyltransferase genes (Sharma et al., 2014) and many others. Recently the genome assemblies of both desi and kabuli chickpeas have been significantly improved and updated (Edwards, 2016a,b). Considering the critical role of NBS-LRR genes in plant defense system against multiple pathogens, it is important to explore this gene family in chickpea and examine their response to ascochyta blight infection. In this study, we identified NBS-LRR gene homologs in the chickpea genome. We analyzed the structural diversity of the chickpea NBS-LRR genes and further classified these genes based on their protein domain architectures. Annotation of the functional domains, phylogenetic tree construction and analysis of genomic distribution of this gene family were completed. We identified co-localized NBS-LRR genes with the previously reported ascochyta blight QTLs (AB-QTLs) and further examined the expression profiles of these co-localized NBS-LRR genes using quantitative real-time PCR (qRT-PCR) at different time points after ascochyta blight infection. This study provides an insight of the NBS-LRR gene family in the chickpea genome and their potential involvement in response to ascochyta blight infection.

## Materials and Methods

### Identification and Classification of Chickpea NBS-LRR Genes

To identify the NBS-LRR genes in the chickpea genome, the genome assembly of CDC Frontier including the predicted gene model annotation was downloaded from NCBI (National Centre of Biotechnology Information^1^). Predicted protein sequences of 28,269 genes in the chickpea genome were initially scanned for the Hidden Markov Model (HMM) profile of NBS/NB-ARC domain (pfam00931) in HMMER v3.1b2 using “hmmsearch” with an expected value (*e*-value) threshold of <1*e*-04. The presence of NBS domain was further confirmed using NCBI conserved domain database (CDD) tool using *e*-value of 0.01 (Marchler-Bauer et al., 2011). The CDD results also confirmed the presence or absence of additional domains such as TIR, CC, and RPW8 in the N-terminal position and a variable number of LRR domains in the C-terminal position. The chickpea NBS-LRR genes were classified based on their protein domain arrangements.

### Identification of Conserved Motifs

The central NBS domain contains several conserved motifs such as P-loop, Kinase-1, GLPL etc. Eight distinct motifs within the NBS domain have been reported in *Arabidopsis* (Meyers et al., 2003). We used a similar approach to identify homologous conserved motifs in NBS domains of chickpea. The protein sequence of NBS domain from each NBS-LRR gene was retrieved and subjected to MEME (Multiple Expectation Maximization for Motif Elicitation; Bailey et al., 2006) for prediction of the conserved motifs.

### Gene Structure, Sequence Alignment, and Phylogenetic Analyses

The exon/intron structure of the chickpea NBS-LRR genes was retrieved from the general feature format (GFF) file of the chickpea genome annotation from NCBI. Multiple sequence alignments were conducted on the full length of the 121 NBS-LRR protein sequences using the default parameters of the Clustal W program. Due to pairwise distance calculation problem, four protein sequences (LOC101489470, LOC105851382, LOC101488657, and LOC101498409) were removed from further analysis. Neighbor-Joining (NJ) phylogenetic tree of 117 protein was constructed with 1,000 bootstrap replications using MEGA7.0. Gene Structure Display Server (GSDS) was used to align the phylogenetic tree and the gene structure of the NBS-LRR genes along with the domain positions.

### Distribution and Cluster Analysis of NBS-LRR Genes

The NBS-LRR genes were distributed across the eight chickpea chromosomes based on the CDC Frontier genome assembly v1. The genes were also mapped on the advanced version CDC Frontier genome assembly v2 for comparison (Edwards, 2016b). To define the gene cluster, the following parameters were established: a cluster must contain at least two genes, the distance between two neighboring NBS-LRR genes should be <200 kb and no more than eight genes between the neighboring NBS-LRR genes.

### Co-localization of NBS-LRR Genes with Ascochyta Blight QTLs

The information of the chickpea AB-QTLs was retrieved from the cool season legume database^2^. The physical locations of the markers associated with AB-QTLs in the chickpea genome were obtained via sequence similarity analysis of both forward and reverse primer sequences of each marker using NCBI BlastN. Only hits with 100% coverage of both query and subject were selected. Based on the physical position of the markers, the physical positions of the corresponding AB-QTLs were inferred on both versions of the CDC Frontier genome assembly. The physical positions of the two candidate genes (CaETR1 and *EIN3*) tagged with QTL_AR1_ and QTL_AR3_ were also retrieved to confirm the physical location of the QTLs. The co-localizations between the NBS-LRR genes and AB-QTLs were analyzed using Microsoft Excel. For visualization, a physical map of the chickpea genome was constructed by combing the distribution of the NBS-LRR genes and the physical location of the AB-QTLs using Mapchart.

### Ascochyta Blight Screening

Three chickpea genotypes including CDC Corinne, CDC Luna (both moderately resistant to ascochyta blight) and ICCV 96029 (susceptible) were used in the greenhouse trial. The experiment was conducted in completely randomized design (CRD). A total of three independent biological replications of each chickpea genotype at each time point under control (non-inoculated) and inoculated were analyzed. Three-week old seedlings were inoculated with single spore derived conidial suspension culture of *Ascochyta rabiei* isolate *AR-170*. About 3 mL of conidial suspension with a concentration of 2 × 10^-5^ conidia mL^-1^ was sprayed on each plant using an air compressor. Control plants were mock-inoculated with water. Following inoculation, all plants were kept under humidity chambers equipped with two humidifiers which maintain relative humidity of 100% for 48 h. Later all plants were moved to greenhouse benches equipped with the overhead misting system and all sides of the bench were covered with plastic sheets to maintain high humidity. Leaf samples were collected at four time points at 12, 24, 48, and 72 hours post inoculation (hpi) from each of the three biological replicates of both control and inoculated plants. Collected tissue samples were immediately frozen in liquid nitrogen and stored at -80°C prior to RNA extraction.

### Quantitative Real-Time PCR (qRT-PCR)

Total RNA was extracted and treated with DNase I using SV Total RNA Isolation kit following manufacturer’s instruction (Promega, USA). Extracted RNA sample quantity was determined by an optical density reading at 260 nm and the OD260/OD280 absorption ratio using NanoDrop 800 UV-vis spectrophotometer (Thermo Fisher Scientific, Inc. USA) and RNA integrity was checked on 1.5% agarose gel electrophoresis. Total RNA (1 μg) was reverse transcribed to cDNA using SensiFAST cDNA synthesis kit (Bioline, Inc.). The cDNA used for qRT-PCR was diluted 5× with DNase/RNase free water. Specific primers were designed for each of the co-localized NBS-LRR genes in AB-QTLs and five reference genes (*18SrRNA*, Elongation factor [*Ef1*α], *GAPDH*, Initiation factor [*IF4a*] and *ACTIN*) using IDT Primer quest tool (Integrated DNA Technologies, Inc). All primer sequences are presented in Supplementary Table 1. The primer pairs were designed to span exon–exon junction with PCR product size between 55–180 bp, the length of primer sequence of 18–25 nucleotides, T*m* between 50–60°C and GC content of 50–60%. Each primer was tested on cDNA and gDNA samples to ensure amplification of the target region. Primer efficiencies of each target and reference gene were calculated by making the 10-fold serial dilution of cDNA using equation (1 + E) = 10 slope (Ramakers et al., 2003). SensiFAST SYBR No-ROX kit was used for the target gene expression using optical 384 well plate on BIO-RAD CFX384 real-time PCR detection system (Bio-Rad laboratories) in accordance with the manufacturer’s protocols. For each gene, two technical replicates of each three biological replication per chickpea genotype at each time point were performed in a single plate along with controls [negative reverse transcription control (-RTC) and no template control (NTC)] for detection of DNA contamination or primer dimers. PCR product specificity of each gene was checked by melting curves analysis carried out by PCR machine after 40 amplification cycles. All experimental samples for each amplicon had a single sharp peak at the amplicon melting temperature.

### qRT-PCR Data Analysis

**FIGURE 1:**
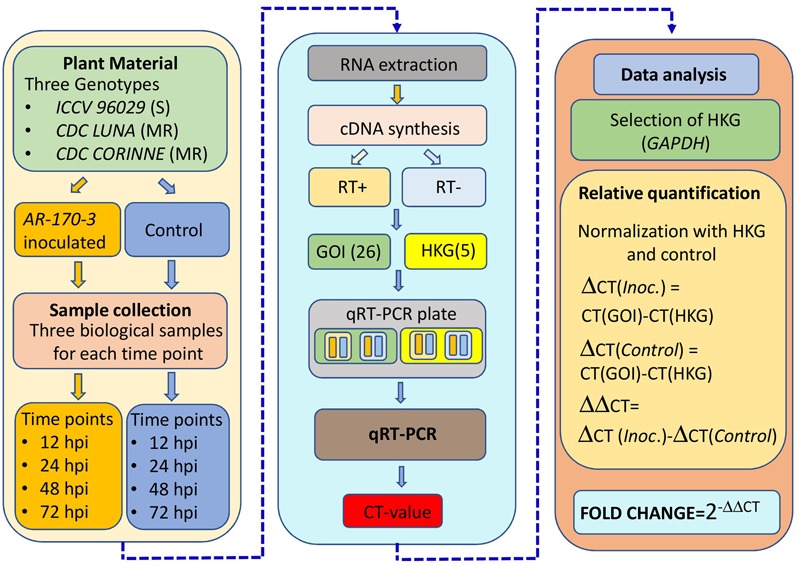
**Overview of the experiment and data analysis for the expression profiling of the co-localized NBS-LRR genes with the known QTLs for ascochyta blight resistance in chickpea**.

Among the tested five reference genes, *GAPDH* was selected and used to normalize the relative quantities of the target genes based on its consistency across the different time points and genotypes. The comparative C_T_ method was used for the quantification of the expression of the co-localized NBS-LRR genes in AB-QTLs in which fold changes in expression were calculated by 2^(-ΔΔCT)^ method (Schmittgen and Livak, 2008). A mean fold change expression level of 2.0 was used as a cut-off point. Differentially expressed genes were clustered using hierarchical cluster analysis. UPMG method was used to generate a dendrogram using *K*-means clustering with Cluster v3.0 program. The heatmap was constructed and displayed using Treeview v1.60. The complete procedure of the expression profiling experiment is summarized in **Figure 1**.

## Results

### Identification and Classification of Chickpea NBS-LRR Genes

We identified a total of 121 NBS-LRR genes in the CDC Frontier genome assembly v1 using our search criteria as explained in the materials and methods. Based on the protein domain combinations, the NBS-LRR genes were grouped into eight classes (**Table 1**). Among the 121 genes, we identified 98 complete genes that carried both the NBS and LRR domains and 23 partial genes that carried the NBS domain but lacked the LRR domain. The majority of the genes belong to the TNL class (39) followed by the CNL class (34) and the NL class (21). We also identified five genes with the RPW8 domain in the N-terminal position other than TIR and CC domain and classified them as the RPW8-NBS-LRR (RNL [4]) and the RPW8-NBS (RN [1]). Sixteen genes that only carried the central NBS domain and lacked both the N-terminal domain and the C-terminal LRRs were classified as the NBS class.

**Table 1:** Classification of the NBS-LRR genes in the chickpea genome.

Set	Class	No. of genes
With LRR	(1) CC-NBS-LRR	34
	(2) TIR-NBS-LRR	39
	(3) RPW8-NBS-LRR	4
	(4) NBS-LRR	21
Without LRR	(5) CC-NBS	3
	(6) TIR-NBS	3
	(7) RPW8-NBS	1
	(8) NBS	16
Total		121

*NBS, Nucleotide Binding Site; LRR, Leucine Rich Repeat; CC, Coiled Coil; TIR, Toll/Interleukin Receptor; RPW8, Resistance to Powdery Mildew 8.*

### Identification of Conserved Motifs within NBS Domain

The MEME motif analysis within the chickpea NBS domain identified eight conserved motifs similar to the *Arabidopsis* NBS domain motif structure. The eight major motifs varied in their divergence within and between the TNL and CNL classes (**Table 2**). Six conserved motifs (P-loop, Kinase-2, RNBS-B, RNBS-C, GLPL and MHDV) were consistently detected in each class. Two motifs RNBS-A and RNBS-D were more diverse in sequence which distinguished the CNL and TNL class. All eight motifs were present in strict order from P-loop to MHDV.

**Table 2:** Consensus sequence of the major motifs identified in the chickpea NBS domain of the CNL and TNL proteins.

Motif	CNL	TNL
*P-loop*	VIPIVGMGGLGKTTLAQLVYND	LGIWGMGGIGKTTLAKAIYNKIXR
*RNBS-A*	DLKAWVCVSDDFDVLKVTKXI	FEGRCFLENVRENSE
*Kinase-2*	LQGKRFLLVLDDVWNEDY	IIKRRLCRKKVLLVLDDVDKLEQ
*RNBS-B*	PCGAKGSKILVTTRNQKVAS	WFGPGSRIIITTRDKHLLXGH
*RNBS-C*	HSLEXLSDEDCWSLFAKHAFR	YEVKELNEKESLELFSWHAFKQDX
*GLPL*	LEKIGKEIVKKCGGLPLAAVT	VVXYAGGLPLALEVLGSFLFGKDI
*RNBS-D*	DKKDLILLWMAEGFL	LDDTEKEIFLDIACF
*MHDV*	FVMHDLVHDLATLVSGEFYFR	MHDLLQDMGREIVREESPKEP

### Sequence Alignment and Phylogenetic Analyses

**FIGURE 2:**
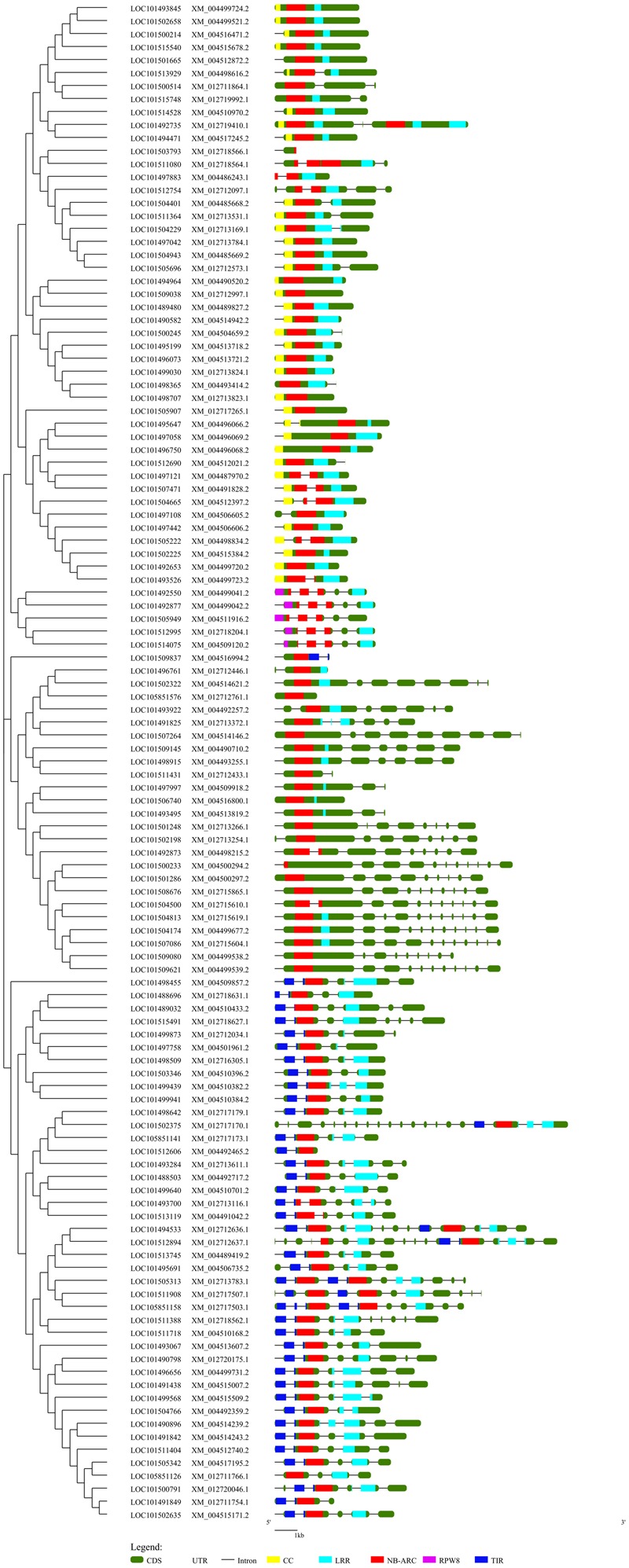
**A Neighbor-Joining phylogenetic tree depicting the evolutionary relationship of the chickpea NBS-LRR genes aligned with the exon-intron structure of each gene along with the domain distribution.** This tree was constructed using 117 complete NBS-LRR protein sequences with 1000 bootstraps. Evolutionary distance was calculated using p-distance method. The gene structure was retrieved from the chickpea annotation and the General Feature File (GFF3). The position of the domains in each gene was obtained from the NCBI conserved domain database (CDD). The phylogenetic tree was aligned with the gene structure along with the domain position using GSDS. Different domains are indicated by different colors.

A NJ phylogenetic tree of 117 complete NBS-LRR proteins was constructed to examine the evolutionary relationships among the NBS-LRR genes (**Figure 2**). Four truncated protein sequences were removed from the analysis after multiple-sequence protein alignment. The NJ tree displayed two clear clades which distinctly separated the TNL class from the non-TNL class. The TNL clade consists of three TNL sub-clades. The non-TNL clades were separated into CNL and NL sister clades. Furthermore, the CNL clade was clearly separated into two sub-clades consisting of CNL clade and RPW8 clade. Phylogenetic clustering of the genes with similar sequences from different chromosomes and the same chromosomes was observed. The alignment of phylogenetic tree with gene structure revealed that exon–intron structure tends to remain the same within the genes present in the same clade reflecting strong conservation of the gene structure during evolution.

### Distribution of the NBS-LRR Genes

**FIGURE 3:**
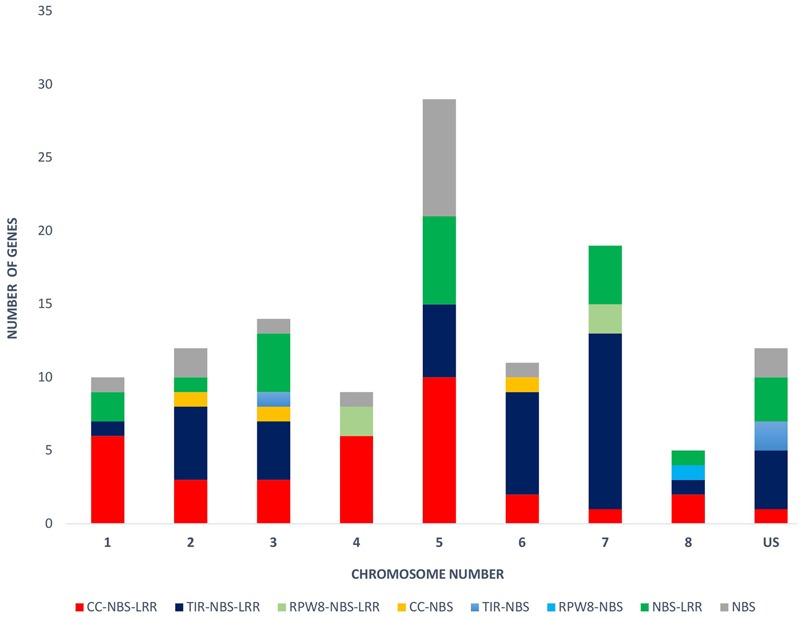
**Distribution of the NBS-LRR genes on each chickpea chromosome (1–8) and unplaced scaffold (US).** Different colors reflect different gene classes.

The physical locations of the NBS-LRR genes were determined based on the chickpea gene annotation and the GFF3 file. Using CDC Frontier genome assembly v1, we were able to place 93 genes on the eight chickpea chromosomes and 28 genes on the unanchored scaffolds. While using the advanced genome assembly version v2, we were able to map 109 NBS-LRR genes on the chickpea chromosomes and 12 were located on the unplaced scaffolds. The chromosomal location of the NBS-LRR genes revealed the uneven distribution of the genes on the eight chickpea chromosomes and showed tandemly located gene clusters (**Figure 3**). Chromosome 5 has the highest number (29) of the NBS-LRR genes (27% of mapped genes), while chromosome 8 has the lowest number (5) of the NBS-LRR genes. At least one CNL gene was present on each chickpea chromosome while the TNL class was limited to seven chromosomes (absent on chromosome 4). Out of the 121 NBS-LRR genes, 58 genes were present in 23 clusters each carrying two to four genes while 68 genes were present as singletons (**Table 3**). Among the 23 clusters, 18 were monophyletic clusters containing 45 genes and 5 were in mixed clusters containing 13 genes. The maximum of four genes per cluster was found in each mono-cluster and mixed-cluster on chromosome 5 and 7, respectively.

**Table 3:** Cluster analysis of the NBS-LRR genes in chickpea.

Cluster type	Cluster	Cluster size (KB)	No. of Genes	Chromosome	Gene ID
Mono-cluster	1	16.4	3	1	LOC101504943, LOC101504401, LOC101505696
	2	3.2	2	2	LOC101513119, LOC101493700
	3	0.8	2	2	LOC101501248, LOC101502198
	4	53.3	3	2	LOC101494533, LOC101512894, LOC101513745
	5	18.9	3	4	LOC101495647, LOC101496750, LOC101497058
	6	71.3	2	4	LOC101495199, LOC101496073
	7	2.9	2	4	LOC101492550, LOC101492877
	8	57.6	3	5	LOC101508676, LOC101500233, LOC101501286
	9	4.4	2	5	LOC101497758, LOC101498509
	10	21.9	2	5	LOC101509621, LOC101509080
	11	144.1	4	5	LOC101507086, LOC101504174, LOC101504500, LOC101504813
	12	45.2	3	5	LOC101492653, LOC101493526, LOC101493845
	13	0.7	2	6	LOC105851141, LOC101502375
	14	40.3	2	6	LOC101511908, LOC105851158
	15	5.6	2	7	LOC101512995, LOC101514075
	16	11.9	3	7	LOC101488696, LOC101515491, LOC101489032
	17	199.2	3	7	LOC101499439, LOC101499941, LOC101503346
	18	20.0	2	7	LOC101490896, LOC101491842
Mixed-cluster	19	116.0	2	1	LOC101511364, LOC101512754
	20	12.0	3	3	LOC101498365, LOC101498707, LOC101499030
	21	18.5	2	5	LOC101497108, LOC101497442
	22	145.1	2	5	LOC101499568, LOC101500514
	23	47.0	4	7	LOC101503793, LOC101511080, LOC101511388, LOC101511718
Total clusters	23		58		
Non-clustered			63		
Total genes			121		

### Co-localization of the NBS-LRR Genes with Ascochyta Blight QTLs

**FIGURE 4:**
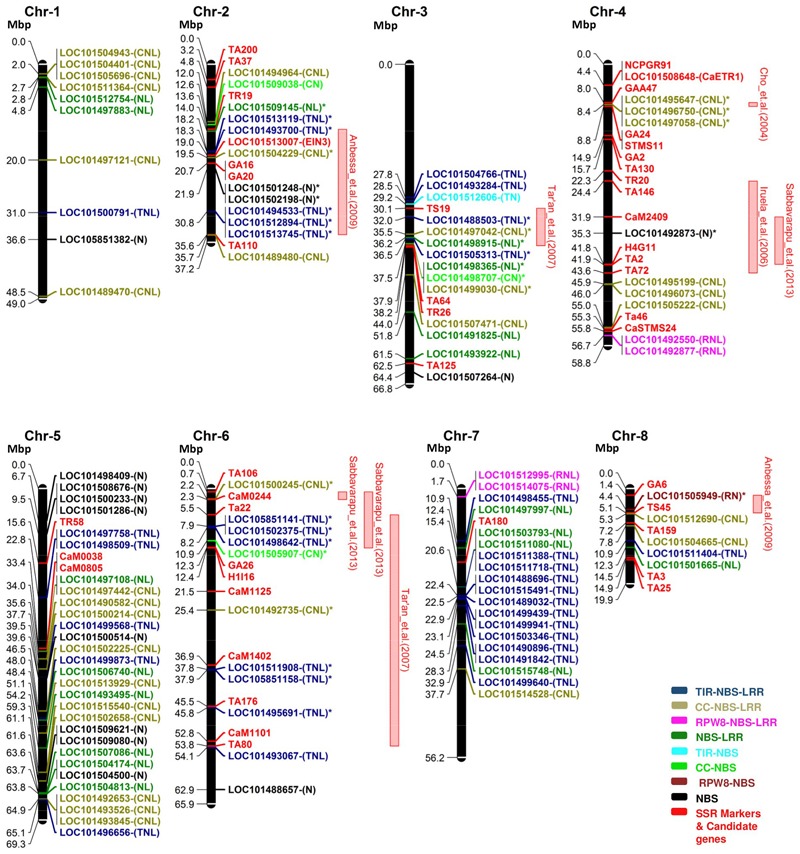
**The distribution of NBS-LRR genes on the physical map of CDC Frontier v2 along with the position of the markers corresponding to the physical positions of the quantitative trait loci (QTLs) for ascochyta blight resistance.** Eight chromosomes (Chr) of chickpea were represented as black bars. Gene and marker names are shown on the right-handed side and their physical positions in megabase pair (Mbp) are shown on the left. Color codes are defined in the legend. QTLs with co-localized NBS-LRR genes were shown as red bars on the right side of the chromosomes. Co-localized NBS-LRR genes are shown with (^∗^) along with their name. We identified 30 NBS-LRR genes co-localized within the nine QTLs for ascochyta blight resistance.

Based on the physical position of the SSR markers on the chickpea chromosomes, 16 QTLs for ascochyta blight resistance previously reported were physically mapped on chromosomes 2, 3, 4, 5, 6 and 8 (Supplementary Table 2). Nine QTLs were co-localized with the NBS-LRR genes. Out of the nine QTLs, three QTLs (Cho et al., 2004; [QTL-AR2] Iruela et al., 2006; [AB-Q-SR-4-2] Sabbavarapu et al., 2013) were mapped on chromosome 4, three QTLs ([QTL4] Tar’an et al., 2007; [AB-Q-APR-6-1, AB-Q-APR-6-2] Sabbavarapu et al., 2013) were mapped on chromosome 6 and one QTL each on chromosome 2 ([QTL1] Anbessa et al., 2009), chromosome 3 ([QTL2] Tar’an et al., 2007) and chromosome 8 ([QTL5] Anbessa et al., 2009). The QTLs ([QTL-AR2] Iruela et al., 2006; [AB-Q-SR-4-2] Sabbavarapu et al., 2013) identified on chromosome 4 in different genetic populations were mapped on the same physical locus. Similarly, QTLs ([QTL4] Tar’an et al., 2007; [AB-Q-APR-6-2] Sabbavarapu et al., 2013) on chromosome 6 were over-lapping. We identified 30 NBS-LRR genes co-located within the flanking markers corresponding to these nine AB-QTLs (**Figure 4**). Among the co-localized NBS-LRR genes, 24 genes were complete genes, i.e., they carry all essential domains for their independent functions. Among these 24 genes, 13 belong to the TNL class, eight belong to the CNL class and three belong to the NL class. Six co-localized genes were incomplete that belong to the RN class (1), CN class (2) and NBS class (3). The majority of the genes (17) co-localized with the AB-QTLs were present in clusters of 2 to 3 genes. On chromosome 2, QTL1 (Anbessa et al., 2009) was co-localized with three mono-clusters; cluster 2, 3 and 4 consisting of two genes from the TNL class, two genes from the NBS class and three genes from the TNL class, respectively (**Table 3**). The QTL2 (Tar’an et al., 2007) on chromosome 3 overlaps with the mixed-cluster 20 consisting of three genes, each from the NL, CN and CNL class. On chromosome 4, the QTL reported by Cho et al. (2004) co-localized with the cluster 5 consisting of three CNL class genes. Two mono-clusters, cluster 13 and cluster 14 each consisting of two genes from the TNL class were co-localized with the AB-Q-APR-6-2 (Sabbavarapu et al., 2013) on chromosome 6.

### Quantitative Real-Time Expression Profiling of the NBS-LRR Genes

**FIGURE 5:**
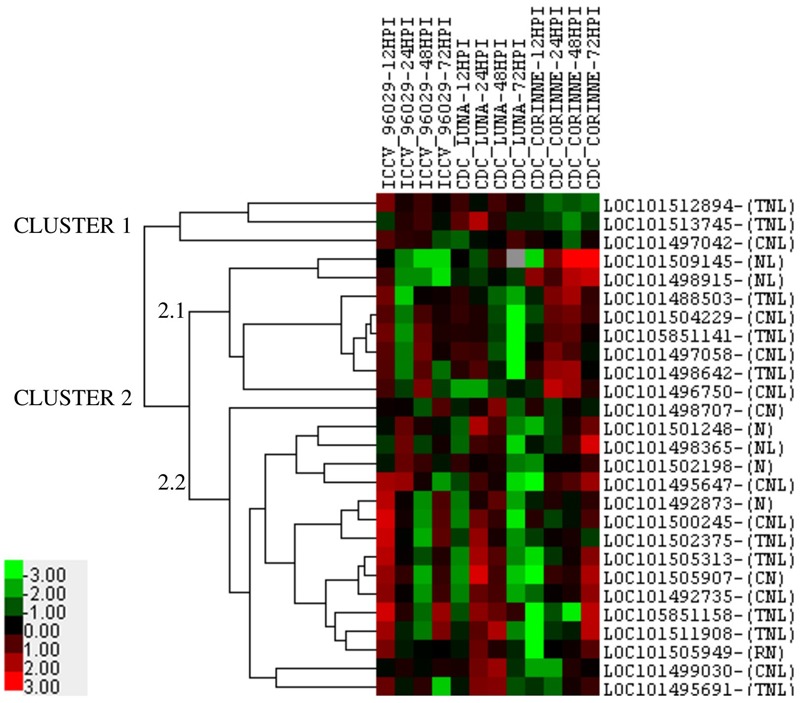
**Heatmap of 27 NBS-LRR genes representing the mean fold change expression levels at four different time points in three chickpea cultivars (ICCV 96029, CDC Luna and CDC Corinne) after infection with *Ascochyta rabiei* isolate *AR-170-3*.** The mean fold change expression values were calculated after normalization with the reference gene (GAPDH) and the non-infected control samples. Red color represents up-regulation, black represents no change and green represents down-regulation as presented in color bar.

Among the 30 co-localized NBS-LRR genes in nine AB-QTLs, 27 genes showed differential expression in response to ascochyta blight infection in at least one genotype at one time point compared to the control (**Figure 5**). The expression of the remaining three genes (LOC101493700, LOC101513119, and LOC101494533) was below the cut-off level of 2.0 mean fold change across all time-points in all genotypes. Five genes showed genotype-specific expression, two genes (LOC101509145, LOC101498915) showed up-regulation only in CDC Corinne at 48 and 72 hpi, and down-regulated or no regulation in ICCV 96029 and CDC Luna at all time points. In contrast, three genes (LOC101512894, LOC101513745, and LOC101497042) showed up-regulation in ICCV 96029 and CDC Luna but no regulation or down-regulated in CDC Corinne. One gene (LOC101505949) constantly expressed in all three genotypes at all time points, except at 12 hpi in CDC Corinne. In terms of the levels of expression, a range of 2–13 mean fold change expression was observed. The highest of 13 mean fold change expression was observed for two genes (LOC101498365 and LOC101511908) in CDC Corinne at 72 hpi when compared to control, followed by 12 mean fold change in three genes; two genes (LOC101505907 and LOC101511908) in CDC Luna and one gene (LOC101500245) in ICCV 96029. Expression profiling of the NBS-LRR genes allowed to differentiate the three genotypes, the susceptible cultivar was separated from the moderately resistant cultivars, and the moderately resistant cultivars were distinguished with respect to the up-regulation of these genes at different time points after inoculation. In ICCV 96029, the highest number of genes (20) were up-regulated at early hours of infection (12 hpi), while 12 NBS-LRR genes were up-regulated in CDC Luna and only 4 NBS-LRR genes were up-regulated in CDC Corinne at 12 hpi. In CDC Luna, the highest number of genes (21) were upregulated at 24 hpi, while 15 and 13 genes were up-regulated in ICCV 96029 and CDC Corinne, respectively. In CDC Corinne, the highest number of NBS-LRR genes (20) were upregulated at both 48 and 72 hpi, while 12 and 18 genes were up-regulated in ICCV 96029 and CDC Corinne, respectively, at 48 hpi, and 14 and 3 genes were up-regulated in ICCV 96029 and CDC Luna, respectively, at 72 hpi. On average, most genes showed up-regulation at 12 and 24 hpi in ICCV 96029, whereas in CDC Luna and CDC Corinne higher expression was observed at 24 and 48 hpi and 48 and 72 hpi, respectively (**Figure 5**).

### Patterns of Gene Expression within and among Genotypes

Cluster analysis of the 27 NBS-LRR genes revealed the underlying expression patterns within and among the genotypes at different time points (**Figure 5**). Two major clusters of expression patterns were observed. Cluster 1 consisted of 3 genes and cluster 2 consisted of 24 genes. Three genes (LOC101512894, LOC101513745, and LOC101497042) in cluster 1 were only up-regulated in ICCV 96029 and CDC Luna, but their expression was below the cut-off limit in CDC Corinne. Cluster 2 consisted of two sub-clusters, cluster 2.1 and cluster 2.2. Eight genes were present in cluster 2.1. Among these, two genes (LOC101509145, LOC101498915) showed contrasting expression pattern to genes present in cluster 1 as they only showed higher expression in CDC Corinne. This suggests that these genes are specific to CDC Corinne in response to ascochyta blight infection. The other six genes in cluster 2.1 were up-regulated in ICCV 96029 at 12 and 48 hpi, in CDC Luna at 12 and 24 hpi and in CDC Corinne at 24 and 48 hpi. The sub-cluster 2.2 consisted of 16 genes, with few exceptions, most genes were up-regulated at 12 and 72 hpi in ICCV 96029, 24 and 48 hpi in CDC Luna, and at 48 and 72 hpi in CDC Corinne.

## Discussion

Ascochyta blight is one of the major yield limiting factor of chickpea production worldwide. However, disease severity is more significant in areas with cooler and wet growing season such as Western Canada (Tar’an et al., 2007). Limited success has been achieved in developing ascochyta blight resistant cultivars due to lack of complete resistance in the chickpea germplasm. To date, several QTLs associated with ascochyta blight resistance have been identified in diverse genetic backgrounds. Yet, the precise mechanism of resistance to ascochyta blight is still unknown.

Numerous plant disease resistance genes including NBS-LRR genes that play a major role in resistance against diverse array of pathogens have been identified and cloned in many plant species (Hammond-Kosack and Jones, 1997). The majority of the NBS-LRR genes were known to provide resistance against biotrophic pathogens following a “gene-for-gene” or “guard” model of host-pathogen interaction leading to the activation of salicylic acid (SA) defense response (Glazebrook, 2005). The knowledge of resistance mechanism against necrotrophic pathogens was limited to phytotoxin production and activation of jasmonic acid (JA) and ethylene pathway (Glazebrook, 2005). An association of the NBS-LRR genes with susceptibility against necrotrophic pathogen has been observed in different studies (Lorang et al., 2007; Nagy and Bennetzen, 2008; Faris et al., 2010). Recent studies also showed the involvement of NBS-LRR genes in resistance reaction against necrotrophic pathogens. For example, *Arabidopsis* TNL class RLM3 gene provides resistance against three necrotrophic fungi; *Botrytis cinerea, Alternaria brassicicola*, and *Alternaria brassicas*, and one hemibiotrophic fungus *Leptosephaeria maculans* (Staal et al., 2008). In wheat, over-expression of the *TaRCR1* gene, a member of CNL class increased resistance against the necrotrophic fungus *Rhizoctonia cerealis* (Zhu et al., 2016).

The involvement of NBS-LRR genes against ascochyta blight infection in chickpea has not been reported so far. Chickpea genome consists of 121 NBS-LRR genes, which is about 0.43% of the total 28,269 annotated genes. Chickpea has relatively low number of NBS-LRR genes compared to *Glycine max* (0.58%), *Medicago truncatula* (0.66%), and *Arabidopsis thaliana* (0.75%; Meyers et al., 2003; Ameline-Torregrosa et al., 2008; Kang et al., 2012). The frequency of NBS-LRR genes is highly variable among plant species, as low as 0.21% in papaya (Porter et al., 2009) and as high as 1.6% in apple (Arya et al., 2014). The chickpea NBS-LRR gene frequency falls within this range. Several studies showed that there is no correlation between the NBS-LRR gene frequency and the genome size or the total annotated genes. One hypothesis for the explanation of the low copy number of NBS-LRR genes might be due to the fitness cost and lethal effect of NBS-LRR genes on plant cells which restrict the number of NBS-LRR genes in plant genome (Zhang et al., 2016). Despite the relatively low number of NBS-LRR genes, most of the chickpea NBS-LRR gene family possess the essential conserved domains as observed in other plant species. Out of the 121 NBS-LRR genes, 98 genes encode proteins consisting of both NBS and LRR domains and the remaining 23 genes were incomplete. The presence of all necessary structural motifs depicts their capacity to function as independent R-proteins. However, the truncated or incomplete genes have also been reported to have a function in co-operation with the complete genes. For example, two tandem NBS-LRR genes; *RPP2A* and *RPP2B* are required to provide resistance against *Peronospora parasitica* isolate Cala2 in *Arabidopsis*. *RPP2A* is incomplete TIR-NBS gene with truncated LRR domain and *RPP2B* is a complete gene, both genes complement each other by providing recognition specificity or signaling lacking by its partner and confer resistance against isolate Cala2 (Sinapidou et al., 2004). Our expression study also showed up-regulation of truncated NBS-LRR genes upon ascochyta blight infection.

Chickpea NBS-LRR gene family were grouped into eight major classes based on their domain architecture. In general, the TNL class is often lacking in monocot species. Chickpea being a dicot species contained TNL class and their numbers (39) were relatively higher than CNL class (34), a pattern similar to *Arabidopsis, Medicago*, soybean and other dicot species. This indicated that the evolution of NBS-LRR genes diversified significantly between monocots and dicots. The evolutionary divergence of TNL from non-TNL/CNL class had been observed in many studies (Meyers et al., 2003; Ameline-Torregrosa et al., 2008; Lozano et al., 2015). It was also observed that RPW8 formed separate sub-clade within CNL clade which supports the functional divergence of the RPW8 from common CNL genes (Collier et al., 2011). Our phylogenetic analysis indicated that chickpea NBS-LRR genes followed the similar patterns. Our analysis showed that among the non-TNL clades, the NL clade was separated from the CNL clade as different clades represent the structural difference among these classes. The phylogenetic analysis supports our criteria of classification into TNL, CNL, RNL and NL and similar classes that lacked the C-terminal LRR domain.

The NBS-LRR genes were distributed across all chickpea chromosomes. However, the distribution was not even across the chromosomes as chromosome 5 contains the highest number of NBS-LRR genes and chromosome 8 has the lowest. Frequently it has been observed that the NBS-LRR genes are present in clusters, which may contribute to the genetic variation and the rapid evolution (Hulbert et al., 2001). In chickpea, nearly half of the NBS-LRR genes (48%) were found in clusters. Among these clustered genes, mono-clusters (78%) were abundant than mixed clusters which reflect that these genes might have evolved through tandem duplications. Another significance of clustering of NBS-LRR genes is that tandem clustering of functionally related genes facilitates co-expression and forms functional heterodimers which might interact with pathogen effector molecules to govern resistance as observed in rice (Ashikawa et al., 2008) and *Arabidopsis* (Sinapidou et al., 2004). Our expression analysis showed similar expression pattern of the NBS-LRR genes present in the cluster. Two NBS-LRR genes (LOC101501248 and LOC101502198) of cluster 3 present in QTL1 (Anbessa et al., 2009) on chromosome 2 showed similar induction pattern in each genotype. Cluster 2 co-located with QTL2 (Tar’an et al., 2007) on chromosome 3, comprising of three NBS-LRR genes (LOC101498365, LOC101498707, and LOC101499030) also showed similar expression pattern in each genotype. Co-expression of these clustered genes reflects their potential involvement in common resistance mechanism.

Co-localized genes in QTL regions have been successfully used to identify potential candidate genes associated with different traits. In chickpea, CaETR1 and *Ein3* have been identified as candidate genes for ascochyta blight resistance based on their co-localization with QTL_AR1_ and QTL_AR3_, respectively, for ascochyta blight resistance (Madrid et al., 2012, 2014). In soybean, the strong positive correlation between the number of NBS-LRR genes and the disease resistance QTLs on each chromosome reflects the contribution of this gene family in soybean disease resistance (Kang et al., 2012). The association of NBS-LRR genes with the ascochyta blight response in chickpea has not been reported. Here, we reported 30 NBS-LRR genes that were co-localized with the physical position of the nine ascochyta blight resistance QTLs on chromosome 2, 3, 4, 6, and 8. Previously it has been reported that cluster of the NBS-LRR genes provides effective resistance in rice and *Arabidopsis* against *Magnaporthe grisea* and *P. parasitica*, respectively (Sinapidou et al., 2004; Ashikawa et al., 2008). Clusters of the NBS-LRR genes were also identified within the AB-QTLs on chickpea chromosome 2, 3, 4 and 6. A cluster of three CNL class genes co-localized with AB-QTL (Cho et al., 2004) on chromosome 4 showed high sequence similarity with the *Arabidopsis* RPP13 gene which provides resistance against five isolates of *P. parasitica* via novel signaling pathway that function independently of SA-mediated response (Bittner-Eddy and Beynon, 2001).

The *Ascochyta rabiei* isolate *AR-170-3* infects both the susceptible and the moderately resistant genotypes as evident by the production of necrotic lesions in all three genotypes. The moderately resistant genotypes (CDC Luna and CDC Corinne) showed delayed symptom development in comparison to the susceptible genotype (ICCV 96029). The majority of the co-localized NBS-LRR genes in AB-QTLs showed differential expression in at least one genotype at one time point compared to control. However, up-regulation of these genes were observed at early hours of infection in the susceptible genotype compared to the resistant genotypes, which correlates with the disease progression on these genotypes. Genotype-specific expression pattern of some of the NBS-LRR genes was also observed. Two NBS-LRR genes (LOC101509145 and LOC101498915) were up-regulated only in the moderately resistant cultivar CDC Corinne. One gene (LOC101505949) co-localized with QTL5 (Anbessa et al., 2009) on chromosome 8 showed up-regulation in all ascochyta blight inoculated samples, except at 12 hpi in CDC Corinne. This gene had very high sequence similarity with the *Arabidopsis ARD1*-like genes (AT4G33300). The *Arabidopsis ARD1*-like gene is an RPW8-NBS-LRR class gene which accumulates SA and provides broad resistance against the biotrophic pathogen (Grant et al., 2003). The presence of biotrophic resistance gene in ascochyta blight resistance QTL suggests common defense mechanism might be involved in providing resistance against biotrophic and necrotrophic pathogens. As cross communication between SA and JA pathway has been previously suggested (Kunkel and Brooks, 2002; Derksen et al., 2013).

Besides the NBS-LRR genes, other disease resistance related genes might also be present within the AB-QTL interval, such as the *EIN3* gene was co-localized with the NBS-LRR genes in QTL2 and QTL_AR3_ (Anbessa et al., 2009; Madrid et al., 2014). *EIN3* is a plant-specific transcription factor which plays an important role in mediating ethylene responses (Madrid et al., 2014). Plant defense response induces several signaling molecules including ethylene, SA and JA which are involved in downstream of the NBS-LRR proteins (McHale et al., 2006). Thus, it is likely that both the ethylene pathway and the NBS-LRR genes might be involved in providing resistance to ascochyta blight. Therefore, it would be interesting to further explore the interaction between the NBS-LRR and the defense responsive ethylene pathway.

In summary, our genetic analysis identified 121 NBS-LRR genes in the chickpea genome. The NBS-LRR genes were classified into eight distinct classes. We identified NBS-LRR genes that are potentially involved in response to ascochyta blight infection based on their co-localization with the known QTLs for ascochyta blight resistance and based on their expression profiles. Our study provides resources for further functional studies to validate the association of NBS-LRR genes with disease resistance in chickpea.

## Author Contributions

MS conducted the experiments, analyzed the data and wrote the manuscript. AD and BT designed the experiment, assisted with data analysis, wrote and edited the manuscript. BT conceived and directed the project. All authors have read and approved the final manuscript.

## Footnotes

1 http://www.ncbi.nlm.nih.gov/assembly/GCF_000331145.1

2 https://www.coolseasonfoodlegume.org
