# Supplementary material for: Genetic Analysis of NBS-LRR Gene Family in Chickpea and Their Expression Profiles in Response to Ascochyta Blight Infection
Source: Front Plant Sci. 2017 May 19;8:838. doi: 10.3389/fpls.2017.00838 (PMC5437156; doi:10.3389/fpls.2017.00838)
Supplement: Supplementary file 1 [file Table_1.DOCX]

Supplementary Table 1. Primer sequence of the co-localized NBS-LRR genes and the reference genes for use in real-time PCR.

| GENE ID | | | Class | PRIMER SEQUENCE | |  |
| --- | --- | --- | --- | --- | --- | --- |
| LOC101493700 | | | *TNL* | F 5'-TAATCAGCATTCTTCTTCTCCATCT-3' | |  |
|  | | |  | R 5'-AACGCCAAATCAAATTCCCTATC-3' | |  |
| LOC105851141 | | | *TNL* | F 5'-GGATGTGCGTCATAAGTCAGA-3' | |  |
|  | | |  | R 5'-GTAGGAGAATCCATCCCAACTAAA-3' | |  |
| LOC101498642 | | | *TNL* | F 5'-GTCCCAACCAACTTGTTGAATTA-3' | |  |
|  | | |  | R 5'-GGGCAAATACTTCTTGTCTTTCC-3' | |  |
| LOC101488503 | | | *TNL* | F 5'-TCAAGAAATGGGCAGGGATATT-3' | |  |
|  | | |  | R 5'-TGCTTCAGTTCCCTTGTTTCT-3' | |  |
| LOC101494533 | | | *TNL* | F 5'-AGGCTCAGAGGATGGAGAA-3' | |  |
|  | | |  | R 5'-CCTGGAGCAATCTGTGAGTATTA-3' | |  |
| LOC101497058 | | | *CNL* | F 5'-GAACTGGAGACCAATCAGAAGG-3' | |  |
|  | | |  | R 5'-GGAGAAACAGGGACAAGATGAG-3' | |  |
| LOC101496750 | | | *CNL* | F 5'-TACGCTCGACGCAATCAATAA-3' | |  |
|  | | |  | R 5'-TCCCAAATTCAGCAGGGAATAG-3' | |  |
| LOC101497042 | | | *CNL* | F 5'-CAACTCACCACCACTCTTCTT-3' | |  |
|  | | |  | R 5'-TGGCCTCTATGTCTTGATTTCTAC-3' | |  |
| LOC101504229 | | | *CNL* | F 5'-CTTCCACCACTTGGACAATTAC-3' | |  |
|  | | |  | R 5'-GGGAGGGAAAGATAATTCCTGA-3' | |  |
| LOC101509145 | | | *NL* | F 5'-TTGAAATTGATAGACCTCCCAAAC-3' | |  |
|  | | |  | R 5'-CAGCAGCAACATCTAATTCTTGA-3' | |  |
| LOC101501248 | | | *NBS* | F 5'-CGGCGTGATAGGTCAGTTAAG-3' | |  |
|  | | |  | R 5'-GCTCTTGTTGCAGCCAAATTA-3' | |  |
| LOC101502198 | | | *NBS* | F 5'-CAGCTGCAAGGTGAGAAGTAT-3' | |  |
|  | | |  | R 5'-GGTTGAATGACCCTGAGTTGTA-3' | |  |
| LOC101505313 | | | *TNL* | F 5'-GGCTTTGTTGGCATGGATTT-3' | |  |
|  | | |  | R 5'-CCCTTCAGAAACTGAGACTTCC-3' | |  |
| LOC101498365 | | | *NL* | F 5'-GGAAGAGAGAGTGACAGGAAAG-3' | |  |
|  | | |  | R 5'-AGCCACCAATTCCCACTATG-3' | |  |
| LOC101498707 | | | *CN* | F 5'-CCGAGCAAGCAAGACATTTG-3' | |  |
|  | | |  | R 5'-ACCCACTCTATCAATGGGAAAC-3' | |  |
| LOC101499030 | | | *CNL* | F 5'-TAGCGGTAGACTCGCATACT-3' | |  |
|  | | |  | R 5'-CAACTCCGAGGACAGGAAATAG-3' | |  |
| LOC101495647 | | | *CNL* | F 5'-GAAAGCACAATGGCAAGGTC-3' | |  |
|  | | |  | R 5'-CAGGGTCGGGTTTGAGAAATA-3' | |  |
| LOC101492873 | | | *NBS* | F 5'-CCTAAGACAGAGAATGCCACAA-3' | |  |
|  | | |  | R 5'-CAACTGTGGTGACTGTGAAGA-3' | |  |
| LOC101500245 | | | *CNL* | F 5'-GCTTCTGAGGAACAACTGGTTA-3' | |  |
|  | | |  | R 5'-ACGGCAAGGTCGTGAATAAG-3' | |  |
| LOC101502375 | | | *TNL* | F 5'-TGATATGCACAAGGTGGATGTAG-3' | |  |
|  | | |  | R 5'-CACCTGAGAAGACGGCATAAA-3' | |  |
| LOC101505907 | | | *CN* | | F 5'-GTTGGCTTTGTCCATGAATCTG-3' | |
|  | |  | | | R 5'-CCCAATCCACCCATACCAATAA-3' | |
| LOC101492735 | | | *CNL* | | F 5'-TGGTCTCCCTCTAGCTGCAA-3' | |
|  | | |  | | R 5'-ACCTTCCCACTCTCTAACATCT-3' | |
| LOC101511908 | | | *TNL* | | F 5'-GGGCAGTGCCTCTGATGTAT-3' | |
|  | | |  | | R 5'-GCTTAGCTCTGCAACTGGCT-3' | |
| LOC105851158 | | | *TNL* | | F 5'-TAGTAGGTTGTGGCGTCCTG-3' | |
|  | | |  | | R 5'-CTTTACAACTTCTGTTCCCTTATGC-3' | |
| LOC101495691 | | | *TNL* | | F 5'-GGATTGCTGGATTGGTGGCT-3' | |
|  | | |  | | R 5'-CCAACTTGACTTTCCCAGGC-3' | |
| LOC101505949 | | | *RN* | | F 5'-TGTCCATGAGATAGTGAGAGGT-3' | |
|  | | |  | | R 5'-GCCACTTCTCAAAAGGCTGC-3' | |
| ACTIN | | | *Act1* | | F 5'-CCTGATGGACAGGTGATCAC-3' | |
|  | | |  | | R 5'-GGAACAGGACCTCTGGACATCT-3' | |
| ELONGATION FACTOR | | | *Ef1α* | | F 5'-TCCACCACTTGGTCGTTTTG-3' | |
|  | | |  | | R 5'-CTTAATGACACCGACAGCAACAG-3' | |
| GAPDH | | | *GAPDH* | | F 5'-CCAAGGTCAAGATCGGAATCA-3' | |
|  | | |  | | R 5'-CAAAGCCACTCTAGCAACCAAA-3' | |
| INITITAION FACTOR | | | *IF4a* | | F 5'-TGGACCAGAACACTAGGGACATT-3' | |
|  | | |  | | R 5'-AAACACGGGAAGACCCAGAA-3' | |
| SMALL RIBOSOMAL RNA | | | *18SrRNA* | | F 5'-ACGTCCCTGCCCTTTGTACAC-3' | |
|  | |  | | | R 5'-CACTTCACCGGACCATTCAAT-3' |  |
