# Supplementary material for: Genetic Analysis of NBS-LRR Gene Family in Chickpea and Their Expression Profiles in Response to Ascochyta Blight Infection
Source: Front Plant Sci. 2017 May 19;8:838. doi: 10.3389/fpls.2017.00838 (PMC5437156; doi:10.3389/fpls.2017.00838)
Supplement: Supplementary file 2 [file Table_2.DOCX]

Supplementary Table 2. List of previously reported QTLs associated with resistance to ascochyta blight and their location on the chickpea physical map of CDC Frontier v2.

| Reference | QTL name |  | Population | Linkage Group | Closest Marker | Chr. | Start (bp) | End (bp) |
| --- | --- | --- | --- | --- | --- | --- | --- | --- |
| Cho et.al.,2004 | - |  | P1359075 x FLIP84-92C | LG2 | TA200 | Ca2 | 3181630 | 3181404 |
|  |  |  |  |  | TA37 | Ca2 | 4794769 | 4794514 |
| Cho et.al.,2004 | - |  | P1359075 x FLIP84-92C | LG2 | GA16 | Ca2 | 20725707 | 20725486 |
|  |  |  |  |  | GA20 | Ca2 | 20725716 | 20725568 |
| Anbessa et.al.,2009 | QTL1 |  | ICCV96029 x CDC-Luna | LG2 | TR13/TR19 | Ca2 | 13605945 | 13606125 |
|  |  |  |  |  | TA110 | Ca2 | 35645481 | 35645662 |
| Tar'an et.al.,2007 | QTL2 |  | ICCV96029 x CDC-Frontier | LG3 | TS19 | Ca3 | 30120220 | 30120309 |
|  |  |  |  |  | TA64 | Ca3 | 37876189 | 37876000 |
| Anbessa et.al.,2009 |  |  | ICCV96029 x AMIT | LG3 | TA64 | Ca3 | 37876189 | 37876000 |
|  |  |  |  |  | TR26 | Ca3 | 38224538 | 38224343 |
| Udupa et.al., 2003 | - |  | ILC1272 x ILC3279 | LG4 | TA130 | Ca4 | 15658926 | 15658717 |
|  |  |  |  |  | TR20 | Ca4 | 22340178 | 22340026 |
| Cho et.al.,2004 | - |  | P1359075 x FLIP84-92C | LG4 | GA24 | Ca4 | 8802287 | 8802468 |
|  |  |  |  |  | GAA47 | Ca4 | 8006877 | 8007024 |
| Sabbavarapu et.al., 2013 | AB-Q-SR-4-2 |  | C214 x ILC3279-F2 | LG4 | CaM2049 | Ca4 | 31877378 | 31573954 |
|  |  |  |  |  | H4G11 | Ca4 | 41777447 | 41777620 |
| Iruela et.al.,2006 | QTL-AR2 |  | ILC3279 x WR315 | LG4 | TA146 | Ca4 | 24367557 | 24367586 |
|  |  |  |  |  | TA72 | Ca4 | 43563684 | 43563874 |
| Madrid et.al., 2012 | QTL_AR1 |  | WR315 x ILC3279 | LG4 | NCPGR91 | Ca4 | 4411683 | 4411370 |
|  |  |  |  |  | GAA47 | Ca4 | 8006877 | 8007024 |
| Sabbavarapu et.al., 2013 | AB-Q-APR-5B |  | C214 x ILC3279 | LG4 | CaSTMS11 | Ca4 | 8802599 | 8802388 |
|  |  |  |  |  | TA130 | Ca4 | 15658926 | 15658717 |
| Sabbavarapu et.al., 2013 | AB-Q-APR-5B |  | C214 x ILC3279 | LG5 | CaM0038 | Ca5 | 33381283 | 33381116 |
|  |  |  |  |  | CaM0805 | Ca5 | 33387179 | 33386904 |
| Sabbavarapu et.al., 2013 | AB-Q-APR-6-1 |  | C214 x ILC3279 | LG6 | TA106 | Ca6 | 685713 | 685506 |
|  |  |  |  |  | H1I16 | Ca6 | 12381876 | 12381715 |
| Sabbavarapu et.al., 2013 | AB-Q-APR-6-2 |  | C214 x ILC3279 | LG6 | TA106 | Ca6 | 685713 | 685506 |
|  |  |  |  |  | CaM0244 | Ca6 | 2274651 | 2274456 |
| Tar'an et.al.,2007 | QTL4 |  | ICCV96029 x CDC-Frontier | LG6 | TA22 | Ca6 | 5494172 | 54941700 |
|  |  |  |  |  | TA80 | Ca6 | 53832720 | 53832897 |
| Anbessa et.al.,2009 | QTL5 |  | ICCV96029 x CDC-Corinne | LG8 | GA6 | Ca8 | 1428366 | 1428214 |
|  |  |  |  |  | TS45 | Ca8 | 5096421 | 5196641 |
